# Adverse Perinatal and Maternal Outcomes and Associated Factors among Women with Antepartum Hemorrhage in Jimma University Medical Center, Southwest Ethiopia, 2020

**DOI:** 10.1155/2022/4594136

**Published:** 2022-08-25

**Authors:** Melkamu Gelan, Tariku Bekela, Kebanesa Angasu, Mosisa Ebisa

**Affiliations:** ^1^Department of Midwifery, Jimma University, Jimma, Ethiopia; ^2^Department of Nursing, Jimma University, Jimma, Ethiopia

## Abstract

**Background:**

Globally, antepartum hemorrhage is the main cause of perinatal and maternal morbidity and mortality during pregnancy and childbearing.

**Objective:**

To assess adverse perinatal and maternal outcomes and associated factors among women with antepartum hemorrhage in Jimma University Medical Center, Southwest Ethiopia, 2020.

**Methods:**

Prospective cross-section study was conducted, and data were collected through face to face interview among pregnant women admitted with antepartum hemorrhage. Patient condition was observed and followed up, and patient card was reviewed. Data were coded, checked, entered into EPI version 4.6, and exported to SPSS version 26.0 for analysis. Multivariate logistic regression analysis was made to determine independent factors associated with adverse outcomes.

**Result:**

A total of 377 pregnant women were included, and nearly half (192 (50.9%)) of women experienced adverse maternal outcome and 113 (30%) women experienced adverse perinatal outcome. The status of vital sign, address, parity, antenatal care, duration of bleeding before arrival, gestational age, prematurity, and amount of vaginal bleeding were factors significantly associated with adverse maternal and perinatal outcome at *p* value <0.05.

**Conclusion:**

Vital sign derangement, vaginal bleeding for ≥12 hrs, gestation age before 37 weeks, rural address, prim parity, amount of vaginal bleeding, and prematurity baby has predicted high rate of adverse perinatal and maternal outcomes. *Recommendation.* Jimma Hospital should give patient-centered service and strengthen counseling on danger sign of pregnancy to alert women early health care seeking and immediate resuscitation, and appropriate management should be given for women admitted with APH to minimize of adverse perinatal and maternal outcomes.

## 1. Introduction

Antepartum hemorrhage is bleeding from the genital canal of the pregnant mother after the fetus has reached the age of viability (which is after 28 completed weeks or fetal weight of 1000gm or more) and before the fetus is delivered [1]. Complications during pregnancy-related death is still high according to WHO recording 295,000, 192,000, and 14,000 maternal deaths worldwide, in Africa, and in Ethiopia in 2017, respectively [2].

Placenta previa, placental abruption, and uterine rupture are main causes of APH. The other cause of APH includestrauma, cancer of cervix (cervical polyps), cervical erosions, endocervical erosions, cervicitis, vulvo-vaginal varicosities, hematuria, vaginitis, foreign bodies, genital lacerations, bloody show, degenerating uterine myomata, vasa previa, and marginal placental separation [1, 3]. Abruption placenta complicates around 1% to 2% of all pregnancies and remains a major contribution of both maternal and fetal morbidity [4].

Majority of women with antepartum hemorrhage deliver low birth weight babies. This is due to preterm labor or repeated small events of hemorrhage causing prolonged placental inadequacy and fetal growth restriction [5]. Antepartum hemorrhage is the main causes of bleeding during pregnancy, maternal and perinatal morbidity and death in developing countries with low capitals and services [6]. Center for Disease Control and Prevention reports that 30% of maternal mortality was due to hemorrhage cases [7]. Antepartum hemorrhage is aggressive situation in obstetrics which complicates about 2–5% of all the pregnancies [8]. Majority of maternal and perinatal morbidity and mortality especially in developing countries with low incomes and resource like Ethiopia was due to obstetric hemorrhage. This is due to well-known pre-existing anemia, problems with transportation, limited health facilities, and lack of responsiveness on part of patient and families which are mainly accountable for great morbidity and death in developing countries. Most of the maternal and perinatal deaths around the world could have been prevented by improving women's contact to good-quality reproductive health care service and effective interventions. So, the purpose of this study is to assess the adverse maternal and perinatal outcome, adverse perinatal and maternal outcomes, and factors associated among women presented with APH in Jimma University Medical Center.

## 2. Methodology

### 2.1. Study Area and Period

The study was conducted in Jimma University Medical Center, which is located in southwest Oromiya, Jimma town, Ethiopia, which is 357 km away from Addis Ababa. This hospital was found in the tertiary level health system. According to 2007 population census projection, Jimma zone has 753,377 women in the child bearing age which accounts 23.1% of all women living in the zone. The labour and maternity ward of Jimma University Medical Center is staffed with 11 obstetrics and gynaecology specialists, 46 midwives, and 33 residents (12 residents of first, 11 residents of second, 6 residents of third, and 6 residents of fourth year), and the study period was conducted from July1, 2020, to June 30, 2021.

### 2.2. Design

Institution-based prospective cross-sectional study design was conducted.

### 2.3. Source Population

All pregnant women admitted with antepartum hemorrhage at Jimma University Medical Center, labor and maternity ward, during this study period.

### 2.4. Study Population

All women presenting with vaginal bleeding after 28 weeks of gestational age, not before the delivery of the fetus in the labor and maternity ward of Jimma University Medical Center.

### 2.5. Measurement

Data were collected by using reviewing patient charts. Patient observation was also made during admission and face-to-face interviewing mothers using a structured and semistructured questionnaire which was used to collect data concerning patient sociodemographic data, obstetric data, causes of APH, and adverse perinatal and maternal outcome.

The questionnaire was prepared in English language and then translated into local language, Afaan Oromo and Amharic, by language experts and translated back to English to sustain reliability. Finally, Afan Oromo and Amharic version was used for data collection. The questionnaire was pretested by taking 5 percent of the sample size at Bedelle Hospital, and required revision was ready depending on gaps identified. Doubled data were entered into EpiData Manager cleaned and explored for outliers, omitted values, and any discrepancies.

### 2.6. Data Collection

The data were collected by three midwives from Jimma university Medical Center staffs by the predesigned questionnaire. Two Obstetrics and Gynacology residents were supervisors of the data collection process. To guarantee the quality of data, two-day training was given for data collectors on the objective and relevance of the study.

### 2.7. Data Analysis

The collected data were coded and entered in to EpiData Manager 4.6 and was transferred to SPSS version 26.0 for analysis. Descriptive and binary logistic regressions analysis was done. These explanatory variables with a *p* value <0.25 in crude analysis were engaged for multivariate analysis, and these variables with a *p* value < 0.05 in multivariate analysis was considered as a significant predictor of maternal complication among women with antepartum hemorrhage. The result of the analysis was presented in texts, tables, and graphs as indicated.

### 2.8. Ethical Clerance

Ethical clearance was obtained from the Jimma University Ethical Review Board (ERB). Approval message to conduct the study was secured from Jimma University Medical Center. Oral informed consents were obtained from respondents in order to execute data collection procedures. The questionnaire was anonymous to maintain privacy and confidentiality among the participants.

### 2.9. Conceptual Definitions

APH: vaginal bleeding that occurs after 28 weeks of gestational age and till the delivery of the fetus. Abraptio placenta: premature separation of normally implanted placenta. Birth weight: weight of the newborn immediately after birth and classified as NBW: 2500–3999gm; LBW: 1500–2499gm; and VLBW: 1000–1499gm. Placenta previa: implantation of the placenta in the lower uterine segment in the zone of cervical dilatation and effacement within 2 cm [9]. Low-lying placenta: When placenta lies inside 2 cm of the cervical opening but does not shield it [9]. Abrupto placenta: premature separation of placenta from normally implanted uterine wall [9].

(1) Mild or grade 1: clinically asymptomatic before delivery and typically detected by the existence of a retro-placental clotting
(2) Moderate or grade 2: midway, the typical marks of abruption, but the fetus is alive
(3) Severe or grade 3: dead fetus and coagulopathy
  - 3A: severe case without coagulopathy
  - 3B: severe case without coagulopathy

### 2.10. Amount of Bleeding [9]

According to Royal College of Obstetrics and Gynaecology guideline, the following definitions have been used:

  Minor hemorrhage: blood loss less than 50 ml has been recognized
  Major hemorrhage: blood loss of 50–1000 ml with no signs of shock
  Massive hemorrhage: blood loss greater than 1000 ml with signs of shock

An adverse perinatal outcome is a composite of one or more of the following: preterm birth (live birth at <37 weeks' gestation), LBW (live birth weighing <2,500 g at birth), stillbirth (fetal deaths occurring >500 g or >22 weeks gestation), early neonatal deaths (neonatal deaths 0–6 days after birth), neonatal deaths (neonatal deaths 0–28 days after birth), perinatal deaths (neonatal deaths 0–6 days plus stillbirths), low APGAR score, and admission to the neonatal intensive care unit (NICU). Diagnosis at NICU and adverse maternal outcome was defined as a composite of one or more of the following: hysterectomy, postpartum hemorrhage (estimated blood loss of >500 mls following vaginal delivery or >1000 mls following cesarean delivery within 24 hours after delivery as documented in the patient's medical registration records and observation), anemia, infection, admission to intensive care unit (ICU), death, and cause of death [10].

## 3. Results

Three-hundred seventy-seven women gave birth in Jimma University Medical Center with a response rate of 98%.

### 3.1. Sociodemographic Characteristics of Adverse Perinatal and Maternal Outcomes

According to the age's distribution, the mean age of pregnant women with antepartum hemorrhage was 27.72 years with a standard deviation of ±7.96 years. Greater than half patient 202(53.6%) were from urban. Among all respondents, 362 (96%) were Oromo and 280 (74.3%) were Muslim by Ethnicity and Religion, respectively. Regarding occupational status, more than half (291 (77.2%)) were housewives. Regarding educational and marital status, 231 (61.3%) could not read and write and 376 (99.7%) were married, respectively (Table 1).

### 3.2. Obstetric History on Adverse Perinatal and Maternal Outcomes

Among patients who admitted with antepartum hemorrhage, most women (255 (67.6%)) were multiparous, greater half of them has no history of abortion (283 (75.1%)) and most of the patients (217 (57.6%)) have ANC follow-up. Majority of women (218 (57.8%)) admitted with APH have came before 12 hrs duration. Most of the women (290 (76.9%)) were admitted with minor amount of vaginal bleeding (Table 2).

### 3.3. Maternal Condition at Admission

A majority of women admitted with APH fetal heart beat (FHB) and at gestational, positive 300 (80%) and term pregnancy 143 (37.9%) respectively. Regarding to initial hematocrit of patient admitted with antepartum hemorrhage majority were 294 (78%) greater than 34%. Majority of women admitted with stable vital sign 349 (92.6%) and on admission 46 (12.2%) admitted with active vaginal bleeding (Table 3).

### 3.4. Cause of APH among Pregnant Women

Regarding the cause of antepartum hemorrhage admitted at Jimma University Medical Center, a majority of women admitted with placenta abruptio and placenta previa were the main reasons of APH which accounts 281 (74.5%) and 77 (20.4%) of APH patients, respectively, and the rest one is unknown (16 (4.2%) and 3 (0.8%)). From those patients with placenta previa, 76 (20.2%) were diagnosed with placenta previa totalis and the rest one had low lying (1 (0.3%)). Of the 281 patients with abruption placenta, over half (159 (56.5%)) diagnosed with moderate or grade 2—midway, the typical marks of abruption; but the fetus is alive.

**Figure 1:**
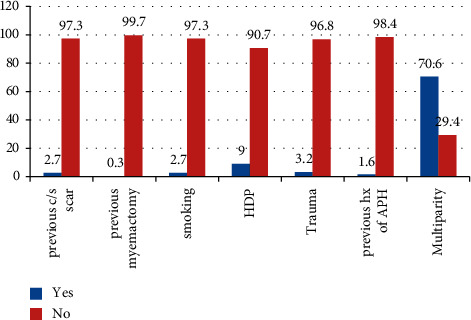
Risk assessment of women admitted with antepartum hemorrhage in Jimma University Medical Center, 2020.

Regarding the risk assessment of patients admitted with antepartum hemorrhage, patient who had multiparity and HDP had greater chance of antepartum hemorrhage (Figure 1).

Regarding the mode of delivery among women admitted with antepartum hemorrhage at Jimma University Medical Center, a majority of them (247 (65.5%)) gave birth SVD, and cesarean section was done in 77 (20.4%) women due to placenta previa (Table 4).

### 3.5. Adverse Perinatal and Maternal Outcomes

From total patients admitted with APH at Jimma University Medical Center, 113 (30%) are prone to more than one adverse perinatal outcome and 192 (50.9%), more than one adverse maternal outcome.

From these adverse maternal outcomes, only five (2.6%) women were referred to intensive care unit (ICU), and at the intensive care unit, 3 (1.6%) women with APH passed away during the peripartum time, two (66.7%) due to eclampsia, and one (33.3%) because of anesthesia-related complication. From women admitted at the maternity and labor ward with antepartum hemorrhage, 44 (24%) developed postpartum hemorrhage.

From these adverse perinatal outcomes in this study, 18 (54.5%) need resuscitation and 32 (94%) were referred to NICU; a majority of them (25 (80%)) were diagnosed with HMD and referred to NICU and 13 (39.45%) died (Table 5).

### 3.6. Fetal Outcome

A total of 393 babies were born from 377 mothers who gave birth at Jimma public Hospitals; of these, 361 (95.8%) were singleton babies; the rest 16 (4.2%) were twins. A majority of babies were delivered at early term (141 (37.4%)). Regarding birth weight, sex, and Apgar score of singleton babies, 246 (65.3%) were of normal weight, 197 (52.3%) were male, and 244 (64.7%) had ≥7 Apgar score, respectively. From a total of 393 babies born, 283 (72%) were live-born and 100 (26.5%) were stillborn (Table 6).

Factors associated with adverse perinatal and maternal outcome among women presented with antepartum hemorrhage in Jimma University Medical Center, 2020.

Bivariate analysis shows that variables like vital sign on admission, previous history of scar, anemia, duration of bleeding before arrival, and mode of delivery with a *p* value <0.25 entered multivariable logistic regression model.

Those variables with *p* value less than 0.05 in multivariate analysis had statistically significant association with adverse perinatal and maternal outcome, and others removed from it. Duration of bleeding before arrival vital sign on admission, gestational age, residence, antenatal follow-up, and parity had strong association with adverse maternal outcome, and prematurity and amount of bleeding had strong association with adverse perinatal outcome. Those mothers who had bleeding ≥12 hrs before arrival to hospital are 2 times more likely prone to adverse maternal outcome than those mothers who arrive less than 12 hours of duration (AOR = 2.21, 95%CI: [1.38, 3.55]). Those mothers whose vital sign was deranged were 4 times more likely to develop adverse maternal outcomes than those mothers with stable vital sign (AOR = 4.93, 95%CI: [2.97, 8.71]). Those term gestational age mothers were 2 times more likely experience adverse maternal outcome than preterm gestational age (AOR = 2.14 95%CI: [1.43, 3.33]). Mothers from rural residence were 2 times more likely to experience adverse maternal outcomes than those from urban (AOR = 2, 95%CI: [1.22, 2.67]). Those patients who has no antenatal follow-up were 2 times more likely to develop adverse maternal outcomes than those who have antenatal follow-up (AOR = 2, 95%CI: [1.30, 2.99]). Those multipara mothers were 2 times more likely develop adverse maternal outcomes than primipara (AOR = 2, 95%CI: [2.33, 2.95]). Those moderate 7 times and severe 4 times the amount of vaginal bleeding were more likely to experience adverse perinatal outcomes than minor amount of vaginal bleeding (AOR = 7, 95%CI: [2.14, 23.10]) and (AOR = 4, 95%CI: [1.22, 14.91]), respectively, and those who had premature baby were 9 times more likely to experience adverse perinatal outcomes than those who does not develop prematurity (AOR = 9, 95%CI: [5.92, 16.52]) (Table 7).

## 4. Discussion

During this study, three-hundred and seventy-seven women gave birth at Jimma University Medical Center. The incidence of antepartum hemorrhage was high 5.5%. But according to a study conducted in Indira Gandhi Medical College, the incidence of APH was found out to be 1.98%, and according to another study conducted in Iraq, the incidence of antepartum hemorrhage was 2.34%. This difference may be due to poor health education about health during antenatal follow-up [5, 11].

Abruption placenta was the top cause of antepartum hemorrhage at Jimma University Medical Center which accounts 281 (74.5%) women. These findings were similar according to a study conducted in South Africa. The primary reason of hemorrhage was abruptio placenta which accounts 37.8% [12]. This study shows that hypertensive disorder during pregnancy and multiparty are risk factors for antepartum hemorrhage. Similar study conducted in India suggesting 73% PIH is one of the key risk factors for APH [13].

From those women diagnosed with antepartum hemorrhage and gave birth in Jimma University Medical Center maternity and labor ward, 192 (50.9%) develop adverse maternal outcome. During this study period, five (2.6%) women referred to intensive care unit (ICU), and at the intensive care unit, 3 (1.6%) women with APH died during the peripartum time, 2 (66.7%) due to eclampsia, and 1 (33.3%) because of anesthesia complication. In this study, among patients admitted with antepartum hemorrhage, 3 (1.6%) patients died, and according to a study conducted in Tertiary Hospital in Ibadan, Nigeria, there were 2 maternal deaths (1%). The difference may be due to low quality of service and care given at Jimma University Medical Center [14].

From total patients admitted with APH at Jimma University Medical Center, 113 (30%) are prone to more than one adverse perinatal outcome. From those experiencing adverse perinatal outcomes in this study, 18 (54.5%) needs resuscitation and 32 (94%) referred to NICU; a majority of them were diagnosed with HMD (25 (80%)) and referred to NICU, and 13 (39.45) died.

The study identified five variables which have strong positive significant associations with adverse maternal outcome. Maternal vital sign on admission, duration of vaginal bleeding before arrival, term, rural, those mothers who has no antenatal follow-up, and multiparty have strong positive association with adverse maternal outcome. Those mothers with deranged vital sign are more likely prone to adverse maternal outcomes than those with stable vital sign, and those patients admitted with moderate and severe vaginal bleeding and prematurity were strongly associated with adverse perinatal outcomes.

## 5. Conclusion and Recommendation

Antepartum hemorrhage affects all pregnancies and increases complication especially in developing countries with low incomes and services and the main causes of maternal complication and death. Duration of bleeding ≥12 hr before arrival, deranged vital sign on admission, term gestational age, patient from rural area, those who has no antenatal follow-up, and multiparty had strong association with adverse maternal outcome, and those patients admitted with moderate and severe vaginal bleeding and prematurity were strongly associated with adverse perinatal outcome. Institution should give inclusive service at labor and delivery unit with well-arranged equipment and staffs with friendly care, timely identification, preparing family for blood donation in case of blood transfusion, skilled anesthesia person, and avail all necessary human resource to effectively manage APH thereby reducing the adverse maternal and perinatal outcome.

## Figures and Tables

**Table 1:** Sociodemographic characteristics of adverse perinatal and maternal outcome and associated factors.

Variable	Category	Frequency	Percent
Age	15–24	145	38.5
	25–34	185	49
	35–49	47	12.5

Address	Urban	202	53.6
	Rural	175	46.4

Ethnicity	Oromo	362	96.0
	Amahara	5	1.3
	Tigray	1	0.3
	Others^*∗*^	9	2.4

Religion	Orthodox	35	9.3
	Protestant	61	16.2
	Muslim	280	74.3
	Others^∗∗^	1	0.3

Occupation	Housewife	291	77.2
	Civil servant(employee)	35	9.3
	Farmer	24	6.4
	Merchant	27	7.2

Educational status	Cannot read and write	231	61.3
	Read and write	64	17.0
	Grade 1–8	41	10.9
	Grade 9–10	4	1.1
	>Grade 10	37	9.8

Marital status	Married	376	99.7
	Unmarried	1	0.3

Income	0–500	5	1.3
	501–1000	49	13.0
	>1000	323	85.7

NB: ethnicity^*∗*^ = Kafa; religion^∗∗^ = Waaqeffanna.

**Table 2:** Obstetric history on adverse perinatal and maternal outcome and associated factors.

Variable	Category	Frequency	Percent
Parity	Primipara	122	32.4
	Multipara	155	67.6

Abortion	Yes	94	24.9
	No	283	75.1

ANC	Yes	217	57.6
	No	160	42.4

Duration of APH before arrival to hospital	<12 hrs	218	57.8
	≥12	159	42.2

Amount of vaginal bleeding	Minor	290	76.9
	Major	73	19.4
	Massive	14	3.7

IV line secured	Yes	202	54
	No	175	46

Patient referred	Yes	178	47.2
	No	199	52.8

Steroid administration	Given	166	44
	Not given	2011	56

**Table 3:** Maternal condition on admission.

Variable	Category	Frequency	Percent
FHB	Positive	300	80
	Negative	77	20

Gestational age	Preterm	230	61
	Term	147	39

Initial hematocrit	≤21%	8	2.1
	22–30%	32	8.5
	31–33%	43	11.4
	>34%	294	78.0

V/s at admission	Stable	349	92.6
	Deranged	28	7.4

Active bleeding on admission	Yes	46	12.2
	No	331	87.8

**Table 4:** Maternal condition at delivery.

Variable	Category	Frequency	Percent
Mode of delivery	SVD	247	65.5
	Operative VD	14	3.7
	Cesarean delivery	116	30.8

If c/s indication (*N* = 116)	CPD	3	0.8
	Failed induction	2	0.5
	Placenta previa	77	20.4
	Malpresentation	3	0.8
	NRFHRP	30	8.0
	Others	2	0.5

**Table 5:** Frequency and distribution of adverse perinatal and maternal outcomes.

	Number	Percent
Adverse maternal outcomes (*N* = 192)		
ICU admission	5	2.6
Is there maternal death?	3	1.6
Cause of death of the mother (*N* = 3)	Eclampsia [2]	66.7
	Anesthesia complication [1]	33.3
Peripartum hysterectomy	3	1.6
PPH	46	24
Anemia	84	44.8
Number of transfusion	24	12.5
Puerperal infection	16	8.3
Long hospital stay	34	17.7

Adverse perinatal outcome		
Prematurity	113	30
Low APGAR (*N* = 377)	33	8.7
Need for resuscitation (*N* = 33)	18	54.5
Referred to NICU (*N* = 33)	31	94
Diagnosis at NICU (*N* = 31)	HMD (*N* = 25)	80
	EONS (*N* = 3)	10
	TTNB (*N* = 3)	10
Neonatal death at NICU	13	39.4

**Table 6:** Fetal outcome of women presented with antepartum hemorrhage in Jimma Zone Public Hospitals, Jimma South West, Ethiopia, 2020.

Variable	Category	Frequency	Percent
Fetal outcome	Single	361	95.8
	Twin	16	4.2

GA of birth outcome	Preterm	166	44
	Term	200	53

Birth weight of single (*N* = 361)	1000gm-1500gm	26	7
	1500–2500	85	24
	2500–4000	250	69

Sex of singleton (*N* = 361)	Male	197	52.3
	Female	164	43.5

APGAR score for single (*N* = 361)	≥7	244	68
	<7	117	32

Weight of twin A (*N* = 16)	1000–1500	1	0.3
	1500–2500	12	3.2
	2500–4000	3	0.8

APGAR score for twin A (*N* = 16)	≥7	14	4
	<7	1	0.3

Weight of twin B (*N* = 16)	1000–1500	3	0.8
	1500–2500	10	2.7
	2500–4000	3	0.8

APGAR of twin B (*N* = 16)	≥7	14	3.7
	<7	1	0.3

Prematurity	Yes	113	30
	No	264	70

Fetal death (SB)	Yes	100	26.5
	No	277	73.5

**Table 7:** Multiple logistic regression analysis: maternal complication among women admitted with antepartum hemorrhage in Jimma University Medical Center, 2020.

Variables		Adverse maternal outcome		COR 95%CI	AOR, 95%CI	*p* value
		Yes	No			
Vital sign	Stable	96	141		1	0.000
	Deranged	17	123	21.3 (10.03, 26.04)	4.93 (2.79, 8.71)	
Duration of vaginal bleeding before arrival	<12 hrs	80	138		1	0.001
	≥12 hrs	33	126	14 (3.35, 61.30)	2.21 (1.38, 3.55)	
GA	Preterm	134	96		1	
	Term	58	89	4.32 (2.31, 10.23)	2.14 (1.43, 3.33)	0.000
Residence	Urban	117	85		1	0.004
	Rural	75	100	5.21 (3.35, 7.36)	2 (1.22, 2.67)	
ANC	Yes	95	122		1	0.001
	No	97	63	7.21 (5.34, 12.56)	2 (1.30, 2.99)	
Parity	Primipara	49	73		1	0.004
	Multipara	143	112	4.78 (3.47, 6.98)	2 (1.33, 2.95)	

Variables		Adverse perinatal outcome		COR 95%CI	AOR 95%CI	*p* value
		Yes	No			
Amount of bleeding	Minor	76	214		1	
	Moderate	27	46	8.8 (3.9, 19.6)	7.04 (2.14, 23.10)	0.001
	Severe	10	4	6.2 (1.5, 24.9)	4.26 (1.22, 14.91)	0.023
Prematurity	No	28	202		1.00	
	Yes	85	62	6.21 (6.34, 8.56)	9.89 (5.92, 16.52)	0.001

NB = *p* < 0.25 (COR); ^∗∗^, =*p* < 0.05 (AOR) significantly associated; 1 = reference.

## Data Availability

Data are available on request.

## Abbreviations

APH: Antepartum hemorrhage
ANC: Antenatal care
FHB: Fetal heart beat
HDP: Hypertensive disorder during pregnancy
JUMC: Jimma University Medical Center
LBW: Low birth weight
SVD: Spontaneous vaginal delivery
WHO: World Health Organization
WCBA: Women of child bearing age.
